# Altered metamemory precedes cognitive impairment in subjective cognitive decline with positive amyloid-beta

**DOI:** 10.3389/fnagi.2022.1046445

**Published:** 2022-10-25

**Authors:** QinJie Li, Feng-Feng Pan, Qi Huang, Chun-Yi Zac Lo, Fang Xie, QiHao Guo

**Affiliations:** ^1^Department of Gerontology, Shanghai Sixth People’s Hospital Affiliated to Shanghai Jiao Tong University School of Medicine, Shanghai, China; ^2^PET Center, Huashan Hospital, Fudan University, Shanghai, China; ^3^Institute of Science and Technology for Brain Inspired Intelligence, Fudan University, Shanghai, China

**Keywords:** subjective cognitive decline, Alzheimer’s continuum, metamemory, amyloid-beta, metacognition, Alzheimer’s disease

## Abstract

Subjective cognitive decline (SCD) as an indicator of preclinical Alzheimer’s disease (AD) may precede mild cognitive impairment (MCI) over several decades. Self-reported cognitive decline as a typical clinical manifestation is critical in preclinical AD. Metacognition represents a person’s ability to accurately assess cognition. Our study aimed to examine (1) the alternations of metamemory in a cohort across the Alzheimer’s continuum, (2) the association between metamemory and cognition, and (3) the relationship of cortical thickness in four regions of interest (ROI) with metamemory scores. Six hundred ninety-seven participants were classified as 79 AD dementia, 161 aMCI, 261 SCD, and 196 cognitively unimpaired (CU) individuals, in which 418 participants aged above 65, 131 participants with Aβ+ after receiving positron emission tomography, and 602 participants received sMRI. The degree of confidence (DOC) was measured by calculating discrepancies between judgments and memory performance. We assessed the relationships between DOC tertiles and cognition and analyzed the screening power, then investigated the partial correlation between DOC and ROIs, controlled by age, sex, and cognition. In the Aβ+ subgroup, SCD showed significantly higher DOC scores than the CU group. There was an increasing trend of overconfidence with the decline of cognition across the AD spectrum (*P* for trend < 0.001). After adjusting for age, sex, and education, the lower degree of confidence-long-term delay recall (DOC-LD) tertiles were associated with lower odds ratio in SCD, aMCI, and AD in the Aβ+ subgroup (all *P* for trend < 0.05). The area under the curves of DOC scores for screening SCD from CU in the Aβ+ subgroup was better than that in all participants and the age ≥65 subgroup. Partial correlation showed that in the Aβ+ subgroup, DOC-SD (degree of confidence-short-term delay recall) was negatively correlated with the anterior cingulate cortex; DOC-LD was negatively correlated with the cortices of parahippocampal, anterior cingulate, posterior cingulate, and medial orbitofrontal. In individuals with Aβ+, SCD exhibited a detectable metamemory alternation before objective cognitive impairment could be tested, indicated by the overestimation in the memory performance. The pattern of an increasing trend of overconfidence across SCD, aMCI, and AD dementia supports the view of a continuum in Alzheimer’s disease.

## Introduction

A self-experienced decline in cognitive capacity, especially in the memory domain, is an important clinical feature in Alzheimer’s disease (AD), amnestic mild cognitive impairment (aMCI), and subjective cognitive decline (SCD) (Albert et al., 2011; Jessen’s et al., 2014). The capacity to accurately assess one’s cognitive states or memory state (metacognition or metamemory) can be impaired in AD and those deficits may become more pronounced as the disease progresses (Starkstein, 2014; Castrillo et al., 2016; Steward et al., 2020; Huntley et al., 2021). Impaired metamemory capacity may portend a preclinical stage of progressive disease and reflect the first effects of AD pathology (Jessen’s et al., 2014; Hanseeuw et al., 2020), such as amyloid-beta (Aβ) burden (Vannini et al., 2017; Hanseeuw et al., 2020) and tauopathy (Vannini et al., 2019). A consensus on the clinical stages of the Alzheimer’s continuum recognized SCD as an indicator of transitional cognitive decline (stage 2) may precede the MCI stage by 10–15 years (Jack et al., 2018), which lies between cognitively unimpaired state (stage 1) and impaired cognition (stage 3) (Jack et al., 2018). The definition of SCD is conceptually dependent on the subjective sense of declined cognition with intact performance on neuropsychological tests (Jessen’s et al., 2014); therefore, the ability to assess and judge a person’s cognitive state may largely influence the accuracy of subjective sense and self-report. However, current knowledge is insufficient regarding the alternation and function of metamemory capacity across the Alzheimer’s continuum, especially in the transitional stage of SCD and with the underpinning of AD pathology.

About 71–93% of patients with AD dementia were observed with metacognition impairment (Starkstein, 2014; Castrillo et al., 2016; Steward et al., 2020; Huntley et al., 2021). Metamemory is described as lacking insight or awareness of their own cognitive and functional impairments in the memory domain (Souchay, 2007). The deficit of metamemory in AD dementia may indicate damage to a personal database or memory storage with the recent information (Stewart et al., 2010; Gagliardi and Vannini, 2022). Previous studies have generally demonstrated impaired metamemory capacity in MCI or mild AD, with patients tending to overestimate their episodic memory performance (Stewart et al., 2010; Dodson et al., 2011; Huntley et al., 2021). Specifically, a general tendency was reported by Fragkiadaki et al. (2016) demonstrating that the overestimation of neuropsychological performance was observed in individuals with aMCI, and underestimation of performance was observed in a group of healthy elderly. Similar patterns of impaired awareness for memory were confirmed by Galeone et al. (2011) in individuals with aMCI and AD, all systematically overestimated on memory neuropsychological tests. In the literature on SCD, the characteristics of metamemory in SCD appear heterogeneous. Recently, a study indicated that metamemory capacity in SCD remains intact, with normal self-awareness of episodic memory performance (Chi et al., 2021). In contrast, another study found that the loss of accuracy in memory monitoring and overestimation of memory performance had already occurred in the SCD stage (Yu et al., 2020). If there is a phenomenon of metamemory deficit in the SCD stage, subjective memory complaints as an important component of clinical diagnosis need further research.

Aβ plaques begin to accumulate decades before the onset of clinical dementia (Jack et al., 2013). In individuals with Aβ burden, low awareness of memory changes may predict clinical progression and increasing levels of clinical impairment in AD (Hanseeuw et al., 2020). Previous studies demonstrated that subjective cognition complaints were more likely correlated with increased amyloid deposition (Perrotin et al., 2012), and memory domain-specific complaints were principally associated with amyloid deposits but not tau pathology in individuals with SCD (Shokouhi et al., 2019). Specifically, higher PiB-PET binding in cognitively normal individuals was primarily associated with subjective memory complaints (Amariglio et al., 2012). Given that episodic memory loss is typically the most prominent clinical feature of AD dementia and is the earliest cognitive domain reported in preclinical AD (Jack et al., 2013), it is plausible to assume that the accuracy of subjective memory complaints is influenced by the metamemory capacity in SCD. Furthermore, the metamemory may change across the Alzheimer’s continuum by the influence of Aβ accumulation at the different stages, possibly due to the damaged memory storage and diminished self-perception of cognition. Whether this type of complaint provides comparable reference value in the preclinical stage of AD in individuals with different underpinnings (i.e., pathology, age) is still a question. Specifically, it remains unclear at which stage in the Alzheimer’s continuum metamemory impairment occurs.

The key regions of neural substrates in the brain associated with metacognition are located in the midline structures, such as the medial prefrontal cortex (mPFC) (Lak et al., 2014; Steward et al., 2019b; Hallam et al., 2020; Huntley et al., 2021), medial temporal lobe (MTL) regions (Hu et al., 2017; Hallam et al., 2020; Huntley et al., 2021), anterior cingulate cortex (ACC), and posterior cingulate cortex (PCC) regions (Fleming and Dolan, 2012; Steward et al., 2019b; D’Oleire Uquillas et al., 2020; Hallam et al., 2020; Huntley et al., 2021), which correlated with the functional connection between self-related (i.e., the mPFC, orbitofrontal) and memory-related (i.e., MTL) networks. Previous studies have consistently demonstrated that decreased gray matter volume in the MTL, such as the hippocampus and parahippocampus (Genon et al., 2016; Vannini et al., 2019), prefrontal cortex (Bertrand et al., 2018), and ACC and PCC (Guerrier et al., 2018; Vannini et al., 2019; Hallam et al., 2020) were associated with impaired metamemory performance in patients with AD dementia and MCI. Individuals who were damaged in the mPFC areas of the brain tended to be overestimated and lose the accuracy of judgments in memory (Modirrousta and Fellows, 2008). The mPFC, especially the medial orbital part (medial orbitofrontal cortex, mOFC), plays an important role in memory monitoring and self-referential processes (Modirrousta and Fellows, 2008; Perrotin et al., 2015). In addition, those regions located in the default mode network (DMN), including the prefrontal cortex, cingulate cortex, and MTL regions (Huntley et al., 2021), are associated with self-related cognitive function and would have connectivity dysfunction with the influence of amyloid-beta and atrophy (Menon, 2011; Weiler et al., 2016). Given the essential role of these regions in metacognition, in the present study, we focused on the relationship of cortical thickness in these areas to metamemory capacity.

Accurate judgments of one’s cognition can have significant consequences for health outcomes, such as seeking medical treatment and making healthcare decisions (Steward et al., 2019a). In assessing metacognition, the focus is on the discrepancy between subjective evaluations and objective performance. The current study seeks to evaluate metamemory capacity by using an established paradigm that requires participants to estimate their episodic memory performance in real-time during the cognitive test, then calculates the results (such as overestimation or underestimation) by the discrepancy between subjective and objective performance. Few studies have explored the metamemory capacity across the Alzheimer’s continuum in individuals in the Chinese population and with Aβ+. The present study aimed to characterize the alternations of metamemory capacity in a cohort of 697 individuals, containing 131 individuals with Aβ+ and 418 individuals aged above 65, across the disease spectrum; moreover, to evaluate the association between metamemory capacity and cognitive stages and provide analyses of the screening efficiency of different cognitive stages for metamemory scores in a Chinese population with two subgroups (Aβ+ and age ≥65); and further investigate the optimal cut-off points according to different subgroups; lastly, to investigate the relationship of cortical thickness in four regions of interest (ROI), including the medial orbitofrontal cortex, parahippocampal, anterior and posterior cingulate cortex, to metamemory scores. Overall, we hypothesized that impaired metamemory capacity may be indicated by overconfidence in memory performance, and the trend would increase with the decline in the cognitive state; furthermore, the altered metamemory capacity may initially present at the stage of SCD in individuals with Aβ+. In addition, the screening efficiency for metamemory scores in individuals with Aβ+ would be better than in individuals with unknowable amyloid-beta; and metamemory scores would be negatively associated with cortical thickness in ROIs.

## Materials and methods

### Participants

A total of 697 participants were recruited between August 2018 and April 2022 from the neuropsychological testing room of Shanghai Sixth People’s Hospital and the community. The inclusion criteria were as follows: educated for more than 6 years; nearly normal eyesight and hearing; with no history of alcoholism, drug abuse, head trauma, history of head surgery, and other neurodegenerative diseases, such as Parkinson’s disease, or neuropsychiatric diseases, such as depression and anxiety. Relevant laboratory tests were carried out to exclude metabolic disorders and nutritional deficiencies, such as abnormalities in folic acid, vitamin B12, thyroid function, and *Treponema pallidum* particle agglutination.

Using the clinical diagnosis, we further subdivided 697 participants into 196 cognitively unimpaired (CU) individuals, 261 SCD, 161 aMCI, and 79 AD dementia. Diagnosis of AD was based on the National Institute of Aging and Alzheimer’s Association criteria (Jack et al., 2011): insidious onset of symptoms and history of cognitive decline by observation. All AD participants were diagnosed with mild AD based on the Mini-Mental State Examination (MMSE) score between 18 and 24. aMCI was mainly based on Jak et al. (2016) and Bondi’s et al. (2014) criteria: significant memory domain impaired scores (defined as >1 standard deviation (SD) below the age-corrected normative mean) on two indexes (AVLT-long-term delay recall and recognition) or memory domain impaired plus any other two cognitive domains impaired scores (defined as >1 SD below the age-corrected normative mean). Individuals with unimpaired cognition were recruited *via* the community, which had a normal performance on standardized cognitive tests used to classify mild cognitive impairment, adjusted for age, sex, and education, and did not meet the criteria of MCI and AD dementia. Individuals with unimpaired cognition who had no subjective cognitive complaints by themselves, and verified by informants, were identified as CU (Jack et al., 2018). Individuals with unimpaired cognition and who met the following criteria were identified as SCD (Jessen’s et al., 2014; Jack et al., 2018): a self-experienced persistent decline in cognitive capacity (decline in memory domain must be included), compared with a previously normal cognitive status, which is unrelated to an acute event. In addition, all SCD must meet more than three features of SCD plus (Jessen’s et al., 2014): age at the onset of subjective cognitive decline ≥60 years; onset of subjective cognitive decline within the last 5 years; feeling of worse performance than others of the same age group; concerns associated with SCD; a confirmed cognitive decline by the informants; and presence of the ApoE e4 genotype and biomarker evidence for a potential progression to AD.

**FIGURE 1:**
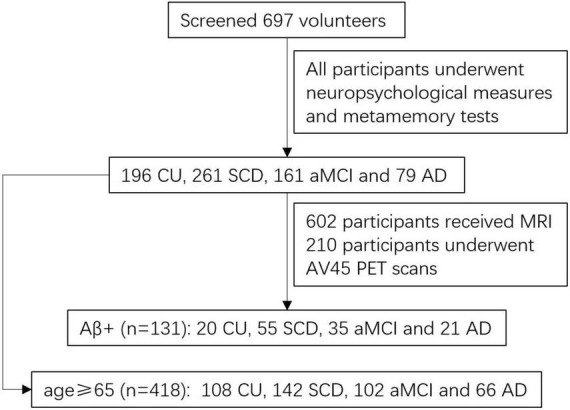
A flowchart of participant selection.

Of the 697 participants, 418 were over 65 years of age, including 108 CU, 142 SCD, 102 aMCI, and 66 AD. Of the total 697 participants, 210 agreed to undergo an AV45 PET scan and 131 reported positive amyloid-beta, including 20 CU, 55 SCD, 35 aMCI, and 21 AD. Of 697 participants, 602 participants received structure magnetic resonance imaging (MRI), including 187 CU, 222 SCD, 132 aMCI, and 61 AD. Figure 1 shows a flowchart of participant selection. The average time between neuropsychological testing and PET/MR imaging was about 3 months. The study was approved by the ethics committee of the Shanghai Sixth People’s Hospital. All participants signed informed consent.

### Neuropsychology assessment

All participants had a comprehensive battery of neuropsychological measures, including general cognitive, domain cognitive (memory, language, and executive function), and metamemory tests. General cognitive tests included Mini-Mental State Examination (MMSE) and Montreal Cognitive Assessment Basic Version (MoCA-B) (Chen et al., 2016). The domain cognitive tests included Auditory Verbal Learning Test-Huashan (AVLT-H) (Zhao et al., 2015), Boston Naming Test (BNT) (Mack et al., 1992), Animal Verbal Fluency Test (AFT) (Zhao et al., 2013a), and Shape Trails Test Parts A (STT-A) and B (STT-B) (Zhao et al., 2013b).

The degree of confidence (DOC) was used to measure the metamemory capacity and embedded in the standardized neuropsychological test for episodic memory (AVLT-H) (Zhao et al., 2015), which includes orally presenting a list of 12 words three times of immediate free recalls, a 5-min short-term delayed recall, a 20-min long-term delayed recall, a cued recall and a recognition. In the process of the AVLT-H, when after the presentation of the whole list and participants finished the three times of free recall, they were required to judge how many words they remembered and predict the words they would remember after 20 min. Those two times of judgments would be recorded, and further, respectively, compared with scores of short-term delay recall (DOC-SD) and long-term delay recall (DOC-LD) using the design formula. DOC was used to evaluate the direction and degree of discrepancies between self-estimated performance (N) and the actual performance (N′), reflecting the extent to which participants were overconfident or unconfident in their performance:


(1) $$\mathrm{DOC}=\frac{\left(12+\left(N-N^{\prime }\right)\right)}{12}$$

### 18F-florbetapir PET acquisition and interpretation

Amyloid PET imaging was used to assess the brain amyloid burden in 131 participants. 18F-florbetapir AV45 tracer was used to quantify amyloid burden (Clark et al., 2012). The PET scans were performed with a PET/CT system (Biograph mCT Flow PET/CT, Siemens, Erlangen, Germany) 50 min after the intravenous injection of 7.4 MBq/kg (0.2 mCi/kg) 18F-florbetapir and lasted for 20 min. PET images were reconstructed by filtered back projection algorithm with corrections for decay, reoriented into a standard 168 × 168 × 148 voxel image grid with 2.04 × 2.04 × 1.5 mm cubic voxels, normalization, dead time, photon attenuation, scatter, and random coincidences. Three board-certified nuclear medicine physicians, including one mid-level and two senior-level titles, visually interpreted all 18F-florbetapir PET images according to visual rating guidelines independently blind to clinical, demographic, and neurological information (Osama Sabri, 2015). The results were based on a consensus of three physicians or a consensus of two.

The qualitative assessment was based on Brain amyloid plaque load (BAPL) scores (1: no amyloid load, 2: minor amyloid load, 3: significant amyloid load) according to the amount of 18F-florbetapir uptake observed on the lateral temporal cortex, frontal cortex, posterior cingulate cortex/precuneus, and parietal cortex (Osama Sabri, 2015; Sabri et al., 2015). Results were interpreted into negative and positive scans (1 = negative scans, 2 and 3 = positive scans) (Osama Sabri, 2015).

### T1-weighted magnetic resonance imaging

Data were collected using a 3.0 Tesla scanner (SIEMENS MAGNETOM, Prisma 3.0T, Siemens, Erlangen, Germany). Three-dimensional T1-weighted images were acquired by using magnetization-prepared rapid gradient-echo sequence in the sagittal plane with the following parameters: matrix = 320 × 320, field of view = 256 mm × 256 mm, slice thickness = 0.8 mm, voxel size = 0.8 mm × 0.8 mm × 0.8 mm, repetition time = 3000 ms, echo time = 2.56 ms, inversion time = 1100 ms, flip angle = 7°, and number of slices = 208. FreeSurfer (v.6.0.0.)^1^ was used to estimate brain cortical thickness. Structural images were automatically processed to reconstruct cortical surfaces and to segment using the FreeSurfer recon-all procedures (see text footnote 1). The procedures were described in previous publications (Fischl et al., 1999, 2002; Fischl and Dale, 2000; Desikan et al., 2006). Briefly, the processing includes motion correction, Talairach transformation, segmentation, topology correction, and normalization. Cortical thickness was obtained by measuring the distance between the pial surface and the white matter boundary. Quality control was conducted by visual assessment and overlapping the parcellations on FreeSurfer’s template. Four regions of interest (ROIs) previously implicated in studies of metamemory were derived based on the Desikan–Killiany atlas, including the cortices of parahippocampal, posterior cingulate, medial orbitofrontal, and anterior cingulate (Guerrier et al., 2018; Steward et al., 2019b; Hallam et al., 2020; Huntley et al., 2021). We chose the mean value of bilateral cortical thickness in this study.

### Statistical analysis

Statistical analyses were conducted using SPSS statistic 23 (IBM, New York, NY, USA). Continuous variables were presented as the mean (standard deviation, SD or standard error, SE), and categorical data were presented as percentages. To evaluate the baseline characteristics by different cognitive states (CU, SCD, aMCI, AD), we used the Mantel–Haenszel test of the trend for categorical variables and linear regression for continuous variables (Girotra et al., 2012). A one-way analysis of covariance (ANCOVA) was performed to test the adjusted mean (i.e., adjusted for the influence of covariates) of metamemory scores among groups, with significantly different demographic characteristics at baseline as covariates (age and education years). Preliminary assumptions were tested to check for normality, linearity, homogeneity of variances, homogeneity of regression slopes, and multi-collinearity. *Post-hoc* tests were used to investigate the between-group differences, with Bonferroni correction of significance levels at *P* < 0.05. Spearman correlation analysis was performed to calculate the relationship between metamemory scores and demographic characteristics, and general neuropsychology test scores. Multivariate logistic regression analysis was conducted to examine the relationship between metamemory scores tertiles and different cognitive states; models adjusted for age, education years, and sex. The receiver operating characteristic curve (ROC) was used to assess the power of metamemory scores as a screening measure to discriminate SCD, aMCI, and AD. The area under the curve (AUC) was used to indicate the diagnostic performance of metamemory scores. Partial correlation was performed to calculate the correlation of metamemory scores and cortical thickness, controlled by age, sex, and groups. A two-tailed *P*-value of less than 0.05 was considered statistically significant.

## Results

### Baseline demographics and standardized neuropsychological tests

Overall, 697 participants included 196 (28.12%) CU, 261 (37.45%) SCD, 161 (23.10%) aMCI, and 79 (11.33%) AD, with 66.86% female subjects, with a mean age of 66.00 ± 7.72 years. Of the total of 697 participants, 418 participants were aged above 65 years old, including 108 (25.84%) CU, 142 (33.97%) SCD, 102 (24.40%) aMCI, and 66 (15.79%) AD, with 61.5% female subjects, with a mean age of 71.06 ± 4.53 years. Of the total 697 participants, 131 participants with a positive Amyloid-beta burden, 20 (15.27%) had CU, 55 (41.98%) had SCD, 35 (26.72%) had aMCI and 21 (16.03%) had AD, with 61.5% female subjects, with a mean age of 65.51 ± 6.82 years.

Supplementary Table 1 describes the trends in demographic and clinical characteristics in participants with different cognitive groups. Overall, there was an increasing trend across four groups with the decline of cognitive states in age in all participants and age ≥65 subgroup (*P* for trend < 0.001 and *P* for trend = 0.002) and a downtrend across four groups with the decline of cognition in education years in all participants, age ≥65 and Aβ+ subgroups (*P* for trend < 0.001, = 0.006 and *P* for trend = 0.045). There was no significance in gender distribution among the four groups. Meanwhile, the results showed that MMSE and MoCA-B, language domain tests (AFT and BNT), and memory tests (long-term delay recall and recognition) appeared to a downtrend with the decline in cognition in all participants, age ≥65 and Aβ+ subgroups (all *P* for trend < 0.001), and STT-A and STT-B appeared an increasing trend with the decline in cognition in all participants, age ≥65 and Aβ+ subgroups (all *P* for trend < 0.001). In addition, the metamemory scores (DOC-SD and LD) showed an increasing trend with the decline in cognition in all participants, age ≥65 and Aβ+ subgroups (*P* for trend < 0.001).

### Between-group comparison of metamemory scores

**FIGURE 2:**
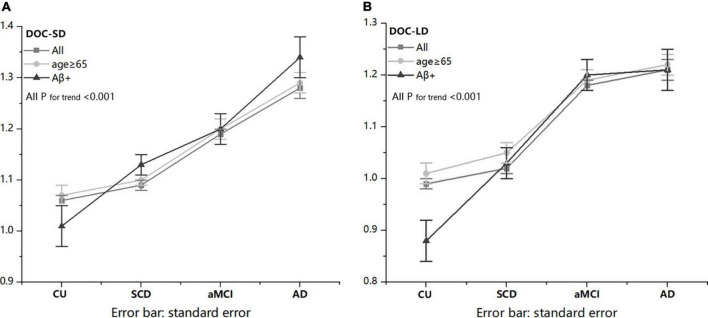
An increasing trend of metamemory scores with the decline of cognitive state in all participants and two subgroups. ANCOVA analyses were performed on the metamemory scores, controlled by the influence of age and education years among four groups.

To control the influence of the imbalanced distribution of demographics among four groups (such as age and education years), the ANCOVA analyses were performed on the metamemory scores, controlled by the age and education years. Results of the ANCOVAs are shown in Table 1 and Figure 2. Preliminary assumptions were tested to check for normality, linearity, homogeneity of variances, homogeneity of regression slopes, and multi-collinearity, with no serious violations noted. Age was evaluated at 66.00 in all participants, 71.06 in age ≥65 subgroup, and 65.51 in the Aβ+ subgroup; education years was evaluated at 11.57 in all participants, 11.27 in age ≥65 subgroup, and 11.47 in the Aβ+ subgroup.

**TABLE 1:** Adjusted means (standard errors) of metamemory scores degree of confidence-short-term delay recall, long-term delay recall (DOC-SD, LD) adjusted for the influence of the covariates^#^.

		CU	SCD	aMCI	AD	*P*
DOC-SD	All	1.06 (0.01)	1.09 (0.01)	1.19 (0.01)^b,d^	1.28 (0.02)^c,e,f^	<0.001
	age ≥65	1.07 (0.02)	1.10 (0.01)	1.20 (0.02)^b,d^	1.29 (0.02)^c,e,f^	<0.001
	Aβ+	1.01 (0.04)	1.13 (0.02)*	1.20 (0.03)^b,d^	1.34 (0.04)*^c,e,f^*	<0.001
DOC-LD	All	0.99 (0.01)	1.02 (0.01)	1.18 (0.01)^b,d^	1.21 (0.02)^c,e^	<0.001
	Age ≥65	1.01 (0.02)	1.05 (0.02)	1.19 (0.02)^b,d^	1.22 (0.02)^c,e^	<0.001
	Aβ+	0.88 (0.04)	1.03 (0.03)^a^	1.20 (0.03)^b,d^	1.21 (0.04)^c,e^	<0.001

^#^ANCOVA analyses were performed on the metamemory scores, controlled by the influence of the imbalanced distribution of age and education years among four groups. *Significant difference between cognitively unimpaired (CU) and subjective cognitive decline (SCD) at *P* < 0.01, uncorrected. ^a^Significant difference between CU and SCD, ^b^Significant difference between CU and amnestic mild cognitive impairment (aMCI), ^c^Significant difference between CU and Alzheimer’s disease (AD), ^d^Significant difference between SCD and aMCI, ^e^Significant difference between SCD and AD, ^f^Significant difference between aMCI and AD, all with *P* < 0.05, Bonferroni correction of significance levels was used in all *post-hoc* tests.

Between groups comparisons of all participants, age ≥65 and Aβ+ subgroups on DOC-SD and DOC-LD were all significant (*P* < 0.001), after controlling for covariates (Figure 2). *Post-hoc* tests showed that aMCI and AD in all participants, age ≥65, and Aβ+ subgroups had higher DOC-SD scores than CU and SCD groups (all *P* < 0.05, Bonferroni-corrected), and the AD group had a further higher DOC-SD scores than aMCI group in all participants, age ≥65 and Aβ+ subgroups (all *P* < 0.05, Bonferroni-corrected). There was no significant difference between CU and SCD on DOC-SD in all participants and age ≥65 subgroup; however, in the Aβ+ subgroup, CU had lower DOC-SD score than that in the SCD group (*P* < 0.01, uncorrected). The DOC-LD in the CU and SCD groups in all participants, age ≥65 and Aβ+ subgroups were significantly lower than aMCI and AD groups (*P* < 0.05, Bonferroni-corrected). In all participants, age ≥65 and Aβ+ subgroups, CU and SCD groups both had significantly lower DOC-LD scores than aMCI and AD groups (*P* < 0.05, Bonferroni-corrected). There was no significant difference between aMCI and AD on DOC-LD scores. In the Aβ+ subgroup, CU had lower DOC-LD score than the SCD group (*P* < 0.05, Bonferroni-corrected).

### Associations between metamemory scores and cognitive function

Spearman correlation analysis (Table 2) revealed that DOC-SD and DOC-LD were positively correlated with age in all participants (*P* < 0.01, *P* < 0.001, respectively) and age ≥65 subgroup (*P* < 0.001, *P* < 0.01, respectively); no correlation relationship was found between metamemory scores and age in Aβ+ subgroups. There was no correlation between sex and education years and metamemory scores. DOC-SD and LD were all negatively correlated with MMSE, MoCA-B, AFT, and BNT in all participants, age ≥65 and Aβ+ subgroup (all *P* < 0.001), except for DOC-SD was negatively associated with AFT and BNT in the Aβ+ subgroup with a significance level at *P* < 0.01. In all participants and age ≥65 subgroup, metamemory scores were positively associated with STT-A, B (all *P* < 0.001). In the Aβ+ subgroup, DOC-LD was positively associated with STT-B (*P* < 0.001).

**TABLE 2:** Spearman correlation of relationship between metamemory scores and age, sex, education years, and neuropsychology test*.

	DOC-SD						DOC-LD					
	All (*n* = 697)		Age ≥65 (*n* = 418)		Aβ + (*n* = 131)		All (*n* = 697)		Age ≥65 (*n* = 418)		Aβ + (*n* = 131)	
	*r*	*p*	*r*	*p*	*r*	*p*	*r*	*p*	*r*	*p*	*r*	*P*
Age	0.110	<0.01	0.177	**< 0.001**	–0.044	0.619	0.146	**< 0.001**	0.127	**< 0.01**	0.084	0.342
Sex	–0.090	0.018	–0.089	0.069	–0.019	0.829	–0.027	0.483	0.006	0.895	0.075	0.397
Education	0.075	0.047	0.055	0.266	0.217	0.013	–0.005	0.896	–0.067	0.173	0.067	0.451
MMSE	–0.335	**< 0.001**	–0.360	**< 0.001**	–0.406	**< 0.001**	–0.356	**< 0.001**	–0.372	**< 0.001**	–0.343	**< 0.001**
MoCA-B	–0.412	**< 0.001**	–0.455	**< 0.001**	–0.481	**< 0.001**	–0.454	**< 0.001**	–0.471	**< 0.001**	–0.469	**< 0.001**
STT-A	0.182	**< 0.001**	0.136	**< 0.001**	0.198	0.023	0.251	**< 0.001**	0.202	**< 0.001**	0.192	0.028
STT-B	0.253	**< 0.001**	0.248	**< 0.001**	0.157	0.078	0.282	**< 0.001**	0.238	**< 0.001**	0.294	**< 0.001**
AFT	–0.159	**< 0.001**	–0.223	**< 0.001**	–0.248	**< 0.01**	–0.216	**< 0.001**	–0.259	**< 0.001**	–0.304	**< 0.001**
BNT	–0.206	**< 0.001**	–0.235	**< 0.001**	–0.257	**< 0.01**	–0.216	**< 0.001**	–0.224	**< 0.001**	–0.293	**< 0.001**

*The level of significance was set at *P* < 0.01 and *P* < 0.001. Bold represents *P* values with significance.

Multivariate logistic regression was performed with the metamemory scores tertiles to estimate the odds ratio (OR) and 95% confidence interval (CI) for the association of metamemory scores with the different cognition stages (Table 3). After adjusting for the age, education years, and sex, the lower DOC-SD tertiles were associated with lower OR in individuals with aMCI and AD in all participants (all *P* for trend < 0.001), age ≥65 subgroup (all *P* for trend < 0.001), and Aβ+ subgroups (aMCI: *P* for trend = 0.006; AD: *P* for trend < 0.001). Lower DOC-LD tertiles were associated with lower OR in SCD, aMCI, and AD in all participants (SCD: *P* for trend = 0.044; aMCI and AD: *P* for trend < 0.001) and Aβ+ subgroup (SCD: *P* for trend = 0.019; aMCI: *P* for trend < 0.001; AD: *P* for trend = 0.003); and in age ≥65 subgroups, lower DOC-LD tertiles were associated with lower OR in individuals with aMCI and AD (all *P* for trend < 0.001), but not in individuals with SCD (*P* for trend = 0.249).

**TABLE 3:** Multivariate logistic regression and adjusted odds ratios (95% CIs) of participants with different cognition groups^a^.

	DOC-SD^b^				DOC-LD^b^			
	T1	T2	T3	*P*_for trend_	T1	T2	T3	*P*_for trend_
**All (697 individuals)**								
	≤1.04	<1.04–≤1.21	>1.21		≤1.00	<1.00–≤1.17	>1.17	
SCD	0.68 (0.42, 1.11)	0.74 (0.44, 1.24)	1.00	0.136	0.57 (0.32, 1.00)	0.71 (0.38, 1.31)	1.00	0.044
aMCI	**0.19 (0.11, 0.33)**	**0.54 (0.32, 0.92)**	1.00	<0.001	**0.08 (0.04, 0.15)**	**0.34 (0.18, 0.61)**	1.00	<0.001
AD	**0.07 (0.03, 0.17)**	**0.21 (0.11, 0.43)**	1.00	<0.001	**0.14 (0.07, 0.28)**	**0.21 (0.10, 0.50)**	1.00	<0.001
**Age ≥65 years (418 individuals)**								
SCD	**0.36 (0.14, 0.94)**	**0.33 (0.13, 0.84)**	1.00	0.144	0.64 (0.32, 1.30)	0.69 (0.32, 1.48)	1.00	0.249
aMCI	**0.06 (0.02, 0.18)**	**0.21 (0.09, 0.52)**	1.00	<0.001	**0.07 (0.03, 0.16)**	**0.36 (0.17, 0.76)**	1.00	<0.001
AD	**0.03 (0.01. 0.08)**	**0.09 (0.03, 0.23)**	1.00	<0.001	**0.13 (0.06, 0.31)**	**0.23 (0.10, 0.54)**	1.00	<0.001
**Aβ+ (131 individuals)**								
SCD	0.21 (0.04, 1.19)	0.36 (0.06, 2.21)	1.00	0.064	**0.11 (0.01, 0.96)**	0.27 (0.02, 3.11)	1.00	0.019
aMCI	**0.08 (0.01, 0.54)**	0.74 (0.04, 1.74)	1.00	0.006	**0.01 (0.00, 0.10)**	0.20 (0.02, 2.31)	1.00	<0.001
AD	**0.01 (0.00, 0.11)**	**0.07 (0.01, 0.54)**	1.00	<0.001	**0.05 (0.01, 0.47)**	0.23 (0.02, 2.96)	1.00	0.003

Bold represents *P* < 0.05. ^a^Models adjusted for the age, education years, and sex; ^b^Metamemory scores were categorized by tertiles.

In all participants, compared to the top tertile of DOC-SD, bottom and middle tertiles in aMCI represented a risk reduction of 81 and 46% (OR: 0.19; 95% CI: 0.11, 0.33 and OR: 0.54; 95% CI: 0.32, 0.92), and bottom and middle tertiles in AD represented a risk reduction of 93 and 79% (OR:0.07; 95% CI: 0.03, 0.17 and OR:0.21; 95% CI: 0.11, 0.43). In all participants, compared to the top tertile of DOC-LD, bottom and middle tertiles in aMCI represented a risk reduction of 92 and 66% (OR: 0.08; 95% CI: 0.04, 0.15 and OR: 0.34; 95% CI: 0.18, 0.61), and bottom and middle tertiles in AD represented a risk reduction of 86 and 79% (OR:0.14; 95% CI: 0.07, 0.28 and OR:0.21; 95% CI: 0.10, 0.50). In age ≥65 subgroup, compared to the top tertile of DOC-SD, bottom and middle tertiles in SCD represented a risk reduction of 64 and 67% (OR: 0.36; 95% CI: 0.14, 0.94 and OR: 0.33; 95% CI: 0.13, 0.84), bottom and middle tertiles in aMCI represented a risk reduction of 94 and 79% (OR: 0.06; 95% CI: 0.02, 0.18 and OR: 0.21; 95% CI: 0.09, 0.52), and bottom and middle tertiles in AD represented a risk reduction of 97 and 91% (OR: 0.03; 95% CI: 0.01, 0.08 and OR: 0.09; 95% CI: 0.03, 0.23). Compared to the top tertile of DOC-LD in age ≥65 subgroup, bottom and middle tertiles in aMCI represented a risk reduction of 93 and 64% (OR: 0.07; 95% CI: 0.03, 0.16 and OR: 0.36; 95% CI: 0.17, 0.76), and bottom and middle tertiles in AD represented a risk reduction of 87 and 77% (OR: 0.13; 95% CI: 0.06, 0.31 and OR: 0.23; 95% CI: 0.10, 0.54). In Aβ+ subgroup, compared to the top tertile of DOC-SD, bottom tertiles in aMCI represented a risk reduction of 92% (OR: 0.08; 95% CI: 0.01, 0.54), and bottom and middle tertiles in AD represented a risk reduction of 99 and 93% (OR: 0.01; 95% CI: 0.00, 0.11 and OR: 0.07; 95% CI: 0.01, 0.54). Compared to the top tertile of DOC-LD in the Aβ+ subgroup, bottom tertiles in SCD represented a risk reduction of 89% (OR: 0.11; 95% CI: 0.01, 0.96), and bottom tertiles in aMCI and AD represented a risk reduction of 99 and 95% (OR: 0.01; 95% CI: 0.00, 0.10 and OR: 0.05; 95% CI: 0.01, 0.47).

### Metamemory scores screening for the subjective cognitive decline, amnestic mild cognitive impairment, and dementia from cognitively unimpaired

**FIGURE 3:**
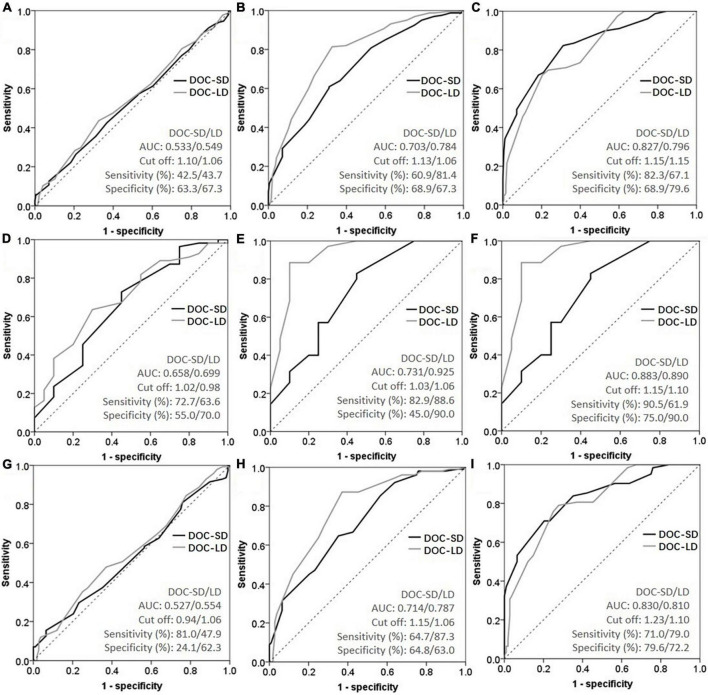
Receiver operating characteristic curve (ROC) curves of metamemory scores in all participants [**(A)** subjective cognitive decline (SCD) vs. cognitively unimpaired (CU); **(B)** amnestic mild cognitive impairment (aMCI) vs. CU; **(C)** Alzheimer’s disease (AD) vs. CU], Aβ+ subgroup [**(D)** SCD vs. CU; **(E)** aMCI vs. CU; **(F)** AD vs. CU], and age ≥65 subgroup [**(G)** SCD vs. CU; **(H)** aMCI vs. CU; **(I)** AD vs. CU].

As shown in Figure 3 and Table 4, we performed ROC curve analysis separately in all participants, Aβ+ and age ≥65 subgroups to evaluate the ability of metamemory scores in discriminating SCD from CU. The optimal cut-off point of DOC-SD for discriminating SCD from CU in all participants was 1.10, with a sensitivity of 42.5%, specificity of 63.3%, and AUC of 0.533 (95% CI, 0.480–0.586); and in the Aβ+ subgroup was 1.02, with a sensitivity of 72.7%, specificity of 55.0%, and AUC of 0.658 (95% CI, 0.541–0.803) and in the age ≥65 subgroup was 0.94, with a sensitivity of 81.0%, specificity of 24.1%, and AUC of 0.527 (95% CI, 0.456–0.599). The most appropriate cut-off point of DOC-LD for discriminating SCD from CU in all participants was 1.06, with a sensitivity of 43.7%, specificity of 67.3%, and AUC of 0.549 (95% CI, 0.496–0.602); and in the Aβ+ subgroup was 0.98, with a sensitivity of 63.6%, specificity of 70.0%, and AUC of 0.699 (95% CI, 0.569–0.829) and in the age ≥65 subgroup was 1.06, with a sensitivity of 47.9%, specificity of 62.3%, and AUC of 0.554 (95% CI, 0.482–0.626), respectively. The AUC of DOC-SD for discriminating aMCI from CU in all participants, Aβ+ and age ≥65 subgroups were 0.703 (95% CI, 0.649–0.757), 0.731 (95% CI, 0.589, 0.874), and 0.714 (95% CI, 0.646, 0.783), respectively, and the AUC of DOC-LD for discriminating aMCI from CU in all participants, Aβ+, and age ≥65 subgroups were 0.784 (95% CI, 0.737–0.831), 0.925 (95% CI, 0.845–1.000), and 0.787 (95% CI, 0.726–0.849). The AUC of DOC-SD for discriminating AD from CU in all participants, Aβ+, and age ≥65 subgroups were 0.827 (95% CI, 0.772–0.882), 0.883 (95% CI, 0.784–0.983), and 0.830 (95% CI, 0.765–0.896), and the AUC of DOC-LD for discriminating AD from CU were 0.796 (95% CI, 0.742–0.851), 0.890 (95% CI, 0.791–0.990), and 0.810 (95% CI, 0.745–0.875) in all participants, Aβ+, and age ≥65 subgroups, respectively. The cut-off point, sensitivity, and specificity are shown in Table 4.

**TABLE 4:** Receiver operating characteristic curve (ROC) analyses for metamemory scores to discriminate subjective cognitive decline (SCD), amnestic mild cognitive impairment (aMCI), and Alzheimer’s disease (AD) from cognitively unimpaired (CU).

Group	Index	AUC	95% CIs	Cut-off	Sensitivity (%)	Specificity (%)
**All**						
SCD vs. CU	DOC-SD	0.533	0.480, 0.586	1.10	42.5	63.3
	DOC-LD	0.549	0.496, 0.602	1.06	43.7	67.3
aMCI vs. CU	DOC-SD	0.703	0.649, 0.757	1.13	60.9	68.9
	DOC-LD	0.784	0.737, 0.831	1.06	81.4	67.3
AD vs. CU	DOC-SD	0.827	0.772, 0.882	1.15	82.3	68.9
	DOC-LD	0.796	0.742, 0.851	1.15	67.1	79.6
**Aβ +**						
SCD vs. CU	DOC-SD	0.658	0.541, 0.803	1.02	72.7	55.0
	DOC-LD	0.699	0.569, 0.829	0.98	63.6	70.0
aMCI vs. CU	DOC-SD	0.731	0.589, 0.874	1.03	82.9	45.0
	DOC-LD	0.925	0.845, 1.000	1.06	88.6	90.0
AD vs. CU	DOC-SD	0.883	0.784, 0.983	1.15	90.5	75.0
	DOC-LD	0.890	0.791, 0.990	1.10	61.9	90.0
**Age ≥65**						
SCD vs. CU	DOC-SD	0.527	0.456, 0.599	0.94	81.0	24.1
	DOC-LD	0.554	0.482, 0.626	1.06	47.9	62.3
aMCI vs. CU	DOC-SD	0.714	0.646, 0.783	1.15	64.7	64.8
	DOC-LD	0.787	0.726, 0.849	1.06	87.3	63.0
AD vs. CU	DOC-SD	0.830	0.765, 0.896	1.23	71.0	79.6
	DOC-LD	0.810	0.745, 0.875	1.10	79.0	72.2

### Partial correlation between metamemory scores and cortical thickness

In the entire cognition groups, a partial correlation between metamemory scores and cortical thickness was performed, controlled by the age, sex, and cognition groups (Table 5). In the Aβ+ subgroup, the DOC-SD was negatively correlated with the anterior cingulate cortex (*r* = −0.401, *P* = 0.021); and the DOC-LD was negatively correlated with the cortices of parahippocampal (*r* = −0.385, *P* = 0.027), posterior cingulate (*r* = −0.409, *P* = 0.018), anterior cingulate (*r* = −0.458, *P* = 0.007), and medial orbitofrontal (*r* = −0.370, *P* = 0.034), respectively. However, there was no significant correlation between metamemory scores and cortical thickness in the age ≥65 subgroup and all participants.

**TABLE 5:** Partial correlation between metamemory scores and cortical thickness.

DOC-SD	All	Age ≥65	Aβ +	DOC-LD	All	Age ≥65	Aβ +
	*r*/*P*	*r*/*P*	*r*/*P*		*r*/*P*	*r*/*P*	*r*/*P*
Parahippocampal	–0.062	–0.094	–0.321		–0.023	–0.007	−**0.385**
	0.129	0.075	0.068		0.567	0.899	**0.027**
Posterior cingulate	–0.013	–0.003	–0.192		–0.012	–0.031	−**0.409**
	0.743	0.949	0.283		0.760	0.563	**0.018**
Anterior cingulate	–0.048	–0.050	−**0.401**		0.008	0.018	−**0.458**
	0.239	0.344	**0.021**		0.853	0.732	**0.007**
Medial orbitofrontal	–0.004	0.011	–0.065		0.047	0.005	−**0.370**
	0.919	0.832	0.717		0.246	0.932	**0.034**

Partial correlation was performed, controlled by age, sex, and cognition groups. Bold represents *P* < 0.05.

## Discussion

The present study aimed to characterize the metamemory in the Chinese population across the Alzheimer’s continuum with positive amyloid-beta (Aβ+), and to evaluate the association between metamemory scores tertiles and cognitive states; further, provide ROC analyses and optimal cut-off points for metamemory scores for different cognition stages; lastly, to investigate the relationship of cortical thickness in four ROIs to metamemory scores.

In general, the neuropsychological measures across CU, SCD, aMCI, and AD, including general cognitive tests and domain scores, all appeared to decrease in performance with the decline of cognitive states. The DOC-SD and LD scores both showed an increasing trend with the decline in cognition in all participants, age ≥65, and Aβ+ subgroups. In addition, we found that in the Aβ+ subgroup, SCD had significantly higher DOC-SD and DOC-LD scores than that in the CU group, and aMCI and AD groups had further significantly higher DOC-SD and DOC-LD scores than the SCD group. DOC scores reflect the degree of overconfidence (value above 1) or under-confidence (value below 1). The trend of metamemory scores across the Alzheimer’s continuum indicated that the level of overconfidence in their episodic memory performance (short-term and long-term delay recall) expanded with the severity of cognitive impairment compared to the CU group in all participants, age ≥65, and Aβ+ subgroups. There was a significantly negative correlation between general neuropsychology tests (MMSE, MoCA-B) and DOC-SD, LD scores. After adjusting for age, sex, and education years, the results showed that lower DOC-LD tertiles were associated with lower OR in individuals with SCD in all participants and Aβ+ subgroup; and lower DOC-SD and DOC-LD tertiles were associated with lower OR in individuals with aMCI and AD in all participants, age ≥65 and Aβ+ subgroups. Furthermore, in the ROC analyses for the identification of SCD, aMCI, and AD from CU using metamemory scores, DOC-SD and DOC-LD scores in the Aβ+ subgroup all showed better AUCs compared with all participants and age ≥65 subgroup.

In this study, we proposed that a slight metamemory deficit may be already present in individuals with Aβ burden in the stage of SCD, indicated by the overconfidence in their memory tests when required participants to make judgments on the numbers they would remember on short-term and long-term delay recalls, though they all had complaints of persistent memory decline in daily life which is unrelated to an acute event and other neuropsychiatric diseases according to Jessen’s et al. (2014, 2020) criterion. However, in participants with unknowable Aβ burden (all participants and age ≥65), there was no difference between CU and SCD in metamemory scores. Unlike a previous study in which proposed metamemory capacity remains intact in individuals with SCD (Chi et al., 2021), we found that SCD tended to be overestimated on their episodic performance in individuals with Aβ burden. The discrepant findings regarding metamemory deficit in SCD in our study and previous study may be due to several causes. For example, the heterogeneity of the population. Chi et al. (2021) did not choose the Aβ+ participants in their study. In groups with unknowable amyloid burden in our study (such as all participants and age ≥65 subgroup), there was also no significant difference between SCD and CU, which is in line with the findings from Chi et al. (2021). In addition, the method of measuring metamemory capacity is also a factor to consider. In their study, Chi et al. (2021) used the visual memory-based global metamemory task, in contrast, we selected the auditory memory-based metamemory test in our study. Aβ plaques as one of the hallmark pathologies of AD begin to accumulate decades before the onset of clinical dementia (Jack et al., 2013) and are correlated with subjective memory complaints in individuals with SCD (Shokouhi et al., 2019). In fact, some degree of self-reported cognitive decline may not be a specific symptom of the preclinical stage of AD; this kind of complaint also could occur in individuals without amyloid pathology. Some previous studies have shown that lower self-awareness can be used as a potential clinical marker of preclinical AD (Cacciamani et al., 2017; Gerretsen et al., 2017). A longitudinal study of 239 elderly people with incident dementia showed that, on average, the awareness of memory function declines 2 to 3 years before dementia onset (Gerretsen et al., 2017). Cacciamani et al. (2017) reported that individuals with SCD who had lower level of awareness showed greater amyloid burden and lower cortical metabolism compared to SCD who had a higher level of awareness. In our study, SCD with Aβ burden exhibited a detectable slight metamemory deficit compared to healthy controls before objective cognitive impairment occurred. In addition, metamemory scores in the Aβ+ subgroup showed more efficacious (higher values of AUCs) for screening SCD from CU compared to groups with unknowable amyloid-beta (all participants and age ≥65 subgroups). It can be speculated that decreased metamemory in SCD may probably serve as a specific clinical indicator of Aβ burden in preclinical AD.

In our study, we found that the severity of metamemory deficit tends to increase as the disease progresses. A negative correlation was found between general neuropsychology tests (MMSE, MoCA-B) and DOC-SD and LD scores and the lower metamemory score tertiles (DOC-LD) were associated with lower OR in different cognitive states (SCD, aMCI, and AD) in the Aβ+ subgroup. This pattern of alternation in AD was consistent with previous studies (Galeone et al., 2011; Berlingeri et al., 2015). Cognitive impairment is often accompanied by disturbed self-awareness capacity in MCI and AD (Modirrousta and Fellows, 2008; Edmonds et al., 2018; Ryals et al., 2019). The personal database (memory storage in the brain) is highly correlated with metacognition and plays a crucial role in the process of self-awareness, which is damaged with the progression of AD disease and influenced by the Aβ accumulation or atrophy at the different stages of the disease (Galeone et al., 2011; Berlingeri et al., 2015). Further, an increasing level of overconfidence in episodic memory with the progression of disease across the Alzheimer’s continuum was reported in our study, which was also consistent with previous findings in MCI and AD, suggesting that overestimation in episodic memory was generally observed (Berlingeri et al., 2015; Fragkiadaki et al., 2016). Interestingly, in cognitively unimpaired elderly people, a slight underestimation of cognitive performance can be observed (Berlingeri et al., 2015; Fragkiadaki et al., 2016). We also found that CU individuals in all participants and the Aβ+ subgroup tended to be under-confident in their memory performance in our study. DOC scores reflect the person to what extent exhibits the degree of overconfidence (value above 1) or under-confidence (value below 1). In Table 1, after controlling the covariance, the DOC-LD in CU was 0.99 ± 0.01 in all participants and 0.88 ± 0.04 in the Aβ+ subgroup (the mean values were all below 1), which indicated a slight underestimation of their actual memory performance when they make judgments. There has been extensive previous work on the characterization of metacognition at the stages of MCI and AD dementia (Starkstein, 2014; Castrillo et al., 2016; Hallam et al., 2020; Huntley et al., 2021); however, our knowledge of which stage in the earlier AD spectrum metamemory deficit occurs is very limited. SCD as an indicator of transitional cognitive decline before the cognitive impairment stage of MCI (Jessen’s et al., 2014), these similar patterns of metamemory characteristics in SCD, aMCI, and AD dementia in our study support the view of a continuum in AD (Jessen’s et al., 2014).

Lastly, we investigated the partial correlations between metamemory scores and cortical thickness in four ROIs, including the cortices of parahippocampal, posterior cingulate, anterior cingulate and medial orbitofrontal, controlling for age, sex, and groups. The results showed that in the Aβ+ subgroup, the DOC-SD was negatively correlated with the cortical thickness of ACC; and the DOC-LD was negatively correlated with the parahippocampal, ACC, PCC, and mOFC, respectively. Previous studies have consistently shown that higher severity of metacognition performance was significantly associated with a decreased volume of gray matter in the MTL (Genon et al., 2016; Vannini et al., 2019), ACC (Cohen et al., 2000; Guerrier et al., 2018), PCC (Genon et al., 2016; Hallam et al., 2020), and mOFC (Cohen et al., 2000; Fleming and Dolan, 2012; Hallam et al., 2020) in AD. The cortical midline structures, including mPFC (including mOFC) (Northoff and Bermpohl, 2004; Perrotin et al., 2015), cingulate cortex (ACC, PCC) (Northoff and Bermpohl, 2004), and MTL (parahippocampal) (Huntley et al., 2021) were the fundamental component of neural basis in metacognition (Perrotin et al., 2015; Huntley et al., 2021). There was intrinsic connectivity among the mPFC, PCC, and hippocampus areas (Weiler et al., 2016; Antoine et al., 2019), which all belong to the default mode network (DMN) and are associated with self-related cognitive activity, such as introspection, autobiographical memory, social function as well as monitoring (Perrotin et al., 2015; Weiler et al., 2016; Antoine et al., 2019; Huntley et al., 2021). Additionally, ACC belongs to the salience network (SN) (Huntley et al., 2021) and has extensive connections between the prefrontal cortex and the rest of the regions of the cingulate cortex (Northoff et al., 2006). It has been demonstrated that AD commonly leads to dysfunction and disconnection in cortical networks with the influence of amyloid pathology and brain atrophy. Alterations in functional connectivity within the DMN and SN are generally observed in AD (Antoine et al., 2019; Hallam et al., 2020). Our findings are in accordance with the most previous knowledge in AD, and these neuroimaging associations support the neural basis of metamemory paradigm in our study. As the results showed that the significant correlations between metamemory scores and ROIs were only reported in the Aβ+ individuals; it is possibly speculated that DOC-SD and DOC-LD are more valuable to individuals with amyloid-beta burden. Future work in larger and longitudinal samples is needed to more comprehensively characterize the metamemory scores, which may map to different brain regions in different cognitive stages in Alzheimer’s continuum.

There are some limitations to this study. First, a longitudinal study needs to be conducted to investigate the presence, evolution, and alternation of these two metamemory scores across the Alzheimer’s continuum in follow-up. Second, the current study only investigates the metacognition capacity based on memory, not considering other cognitive domains (language, executive function). Further studies need to compare metamemory to other domain-specific metacognition. Third, the current study only considered brain amyloidosis as a neuropathological event in AD. Studies exploring the relationships between tau or peripheral blood markers and metamemory should be conducted in future.

In the current study, we found that in the Aβ+ subgroup, SCD exhibited a detectable metamemory deficit compared to the CU group. The trend of metamemory scores across the Alzheimer’s continuum indicated an increasing level of overconfidence in episodic memory performance with the decline of cognition. We confirmed that the lower metamemory scores were associated with lower odds ratio in individuals with SCD in the Aβ+ subgroup. Furthermore, in the Aβ+ subgroup, higher metamemory scores were correlated with thinner cortices in the midline brain areas. This is the first study to characterize the features of metamemory capacity in the Chinese population across the Alzheimer’s continuum. The current analysis is part of an ongoing large-scale multimodal imaging study. Future investigations will include larger samples and include other modalities, such as blood-based biomarkers.

## Data availability statement

The datasets presented in this article are not readily available because the current analysis is part of an ongoing large-scale study. Requests to access the datasets should be directed to QHG, qhguo@sjtu.edu.cn.

## Ethics statement

The studies involving human participants were reviewed and approved by Shanghai Sixth People’s Hospital. The patients/participants provided their written informed consent to participate in this study.

## Author contributions

QJL and QHG contributed to the study conception and design. QJL, F-FP, QH, C-YL, and FX contributed to the data collection and interpretation. QJL wrote the first draft of the manuscript. QHG contributed to the critical revision of the manuscript and was the guarantor for this study. All authors contributed to the article and approved the submitted version.

## Footnotes

1
http://surfer.nmr.mgh.harvard.edu
