# Supplementary material for: Altered metamemory precedes cognitive impairment in subjective cognitive decline with positive amyloid-beta
Source: Front Aging Neurosci. 2022 Oct 25;14:1046445. doi: 10.3389/fnagi.2022.1046445 (PMC9640736; doi:10.3389/fnagi.2022.1046445)
Supplement: Supplementary file 1 [file Table_1.DOCX]

Supplementary Material

**Supplementary Table 1.** Trends in demographics and neuropsychological tests in participants with different cognitive states.

| **All/age≥65/Aβ+** n=697/418/131 | | **CU** | **SCD** | **aMCI** | **AD** | **P for trend** |
| --- | --- | --- | --- | --- | --- | --- |
|  |  | 196/108/20 | 261/142/55 | 161/102/35 | 79/66/21 |  |
| **Age** | All | 65.61 (7.39) | 64.65 (7.72) | 66.45 (7.54) | 70.51 (7.29) | <0.001 |
|  | age≥65 | 70.89 (4.62) | 70.26 (4.16) | 71.19 (4.30) | 72.86 (5.05) | 0.002 |
|  | Aβ+ | 67.65 (6.89) | 64.33 (6.14) | 65.09 (7.32) | 67.29 (7.12) | 0.816 |
| **Female (%)** | All | 67.86 | 67.05 | 66.46 | 64.56 | 0.604 |
|  | age≥65 | 61.11 | 64.79 | 57.84 | 60.61 | 0.660 |
|  | Aβ+ | 60.00 | 56.36 | 60.00 | 66.67 | 0.561 |
| **Education** | All | 12.24 (3.05) | 11.74 (3.25) | 11.23 (3.36) | 10.07 (4.41) | <0.001 |
| **(years)** | age≥65 | 11.80 (3.26) | 11.32 (3.18) | 11.28 (3.44) | 10.24 (4.48) | 0.006 |
|  | Aβ+ | 12.53 (3.06) | 11.70 (3.38) | 11.14 (3.41) | 10.38 (4.78) | 0.045 |
| **MMSE** | All | 28.25 (1.65) | 27.57 (1.75) | 26.70 (1.88) | 20.95 (3.29) | <0.001 |
|  | age≥65 | 28.11 (1.79) | 27.35 (1.68) | 26.65 (1.69) | 21.11 (2.69) | <0.001 |
|  | Aβ+ | 28.20 (1.77) | 27.75 (1.82) | 26.26 (2.05) | 21.14 (2.90) | <0.001 |
| **MoCA-B** | All | 26.08 (2.41) | 24.69 (3.21) | 21.88 (3.58) | 15.27 (4.03) | <0.001 |
|  | age≥65 | 25.81 (2.58) | 24.10 (3.36) | 21.57 (3.23) | 15.30 (3.74) | <0.001 |
|  | Aβ+ | 26.70 (1.87) | 24.73 (3.12) | 21.77 (3.90) | 16.05 (4.39) | <0.001 |
| **STT-A** | All | 46.82 (16.00) | 49.15 (16.46) | 60.28 (26.34) | 92.18 (59.65) | <0.001 |
|  | age≥65 | 49.43 (16.78) | 52.75 (17.71) | 63.98 (28.16) | 87.71 (48.15) | <0.001 |
|  | Aβ+ | 44.35 (10.04) | 46.69 (13.40) | 63.86 (37.11) | 95.38 (40.74) | <0.001 |
| **STT-B** | All | 123.19 (37.22) | 124.98 (37.60) | 155.01 (51.48) | 193.45 (54.81) | <0.001 |
|  | age≥65 | 131.36 (38.89) | 136.14 (36.77) | 163.01 (53.20) | 193.48 (52.99) | <0.001 |
|  | Aβ+ | 128.65 (52.67) | 115.84 (28.03) | 156.88 (51.58) | 174.50 (62.73) | <0.001 |
| **AFT** | All | 17.88 (4.16) | 15.92 (3.91) | 13.38 (4.37) | 11.05 (5.16) | <0.001 |
|  | age≥65 | 17.87 (4.15) | 15.69 (4.17) | 13.38 (4.56) | 11.28 (5.39) | <0.001 |
|  | Aβ+ | 19.17 (3.11) | 15.96 (4.12) | 13.53 (3.55) | 10.84 (3.20) | <0.001 |
| **BNT** | All | 24.44 (3.12) | 23.93 (3.37) | 20.96 (4.35) | 18.21 (4.84) | <0.001 |
|  | age≥65 | 24.75 (2.94) | 23.84 (3.47) | 21.48 (4.15) | 18.35 (4.94) | <0.001 |
|  | Aβ+ | 24.88 (3.08) | 24.30 (3.60) | 21.74 (4.65) | 17.60 (5.70) | <0.001 |
| **Long-term delay recall** | All | 5.94 (2.44) | 4.83 (2.63) | 2.14 (1.94) | 0.42 (0.89) | <0.001 |
|  | age≥65 | 5.31 (2.26) | 4.26 (2.68) | 1.69 (1.64) | 0.30 (0.68) | <0.001 |
|  | Aβ+ | 6.85 (2.30) | 4.67 (2.78) | 1.77 (1.73) | 0.29 (0.56) | <0.001 |
| **Recognition** | All | 21.91 (2.01) | 21.75 (1.54) | 17.80 (2.73) | 14.95 (3.22) | <0.001 |
|  | age≥65 | 21.71 (1.74) | 21.40 (1.55) | 17.30 (2.55) | 14.74 (3.13) | <0.001 |
|  | Aβ+ | 22.25 (1.59) | 21.80 (1.57) | 17.57 (2.44) | 15.10 (3.02) | <0.001 |
| **DOC-SD** | All | 1.06 (0.16) | 1.09 (0.18) | 1.19 (0.17) | 1.28 (0.16) | <0.001 |
|  | age≥65 | 1.07 (0.16) | 1.09 (0.18) | 1.20 (0.16) | 1.29 (0.16) | <0.001 |
|  | Aβ+ | 1.02 (0.21) | 1.14 (0.17) | 1.20 (0.17) | 1.33 (0.16) | <0.001 |
| **DOC-LD** | All | 0.99 (0.19) | 1.02 (0.20) | 1.18 (0.17) | 1.21 (0.19) | <0.001 |
|  | age≥65 | 1.01 (0.19) | 1.05 (0.19) | 1.19 (0.16) | 1.23 (0.18) | <0.001 |
|  | Aβ+ | 0.89 (0.18) | 1.03 (0.21) | 1.20 (0.13) | 1.21 (0.22) | <0.001 |

***: P<0.001. MMSE, Mini-Mental State Examination; MoCA-B, Montreal Cognitive Assessment-B version; STT, Shape Trails Test Parts; AFT, Animal Verbal Fluency Test; BNT, Boston Naming Test, DOC-SD, degree of confidence-short-term delay recall, DOC-LD, degree of confidence-long-term delay recall.
